# Gonadal dysfunction in a man with Noonan syndrome from the *LZTR1* variant: case report and review of literature

**DOI:** 10.3389/fendo.2024.1354699

**Published:** 2024-04-16

**Authors:** Francesca Orsolini, Luisa Pignata, Fulvia Baldinotti, Silvia Romano, Massimo Tonacchera, Domenico Canale

**Affiliations:** ^1^ Department of Clinical and Experimental Medicine, Endocrine Unit, University of Pisa, Pisa, Italy; ^2^ Department of Laboratory Medicine, Section of Molecular Genetics, Pisa University Hospital, Pisa, Italy; ^3^ Departmental Section of Medical Genetics, Pisa University Hospital, Pisa, Italy

**Keywords:** Noonan syndrome, gonadal function, fertility, LZTR1 variant, case report

## Abstract

Noonan syndrome (NS) is a genetic disorder characterized by multiple congenital defects caused by mutations in the RAS/mitogen-activated protein kinase pathway. Male fertility has been reported to be impaired in NS, but only a few studies have focused on fertility status in NS patients and underlying mechanisms are still incompletely understood. We describe the case of a 35-year-old man who underwent an andrological evaluation due to erectile dysfunction and severe oligospermia. A syndromic facial appearance and reduced testis size were present on clinical examination. Hormonal evaluation showed normal total testosterone level, high FSH level, and low–normal AMH and inhibin B, compatible with primary Sertoli cell dysfunction. Genetic analysis demonstrated the pathogenetic heterozygous variant c.742G>A, p.(Gly248Arg) of the *LZTR1* gene (NM_006767.3). This case report provides increased knowledge on primary gonadal dysfunction in men with NS and enriches the clinical spectrum of NS from a rare variant in the novel gene *LZTR1*.

## Introduction

Noonan syndrome (NS) is a relatively common genetic disorder characterized by multiple congenital defects with an estimated incidence of one in 1,000 to 2,500 live births (1). It has been classically considered an autosomal dominant disorder with variable expressivity, but most cases are sporadic (*de novo*), and recently, cases with autosomal recessive inheritance have been described (2, 3). It is caused by variants in the RAS/mitogen-activated protein kinase (MAPK) pathway involved in cell proliferation, differentiation, and survival (4). Approximately 50% of individuals with NS have a pathogenic missense variant in the *PTPN11* gene, which encodes the non-receptor protein tyrosine phosphatase SHP-2. In the remaining cases (25%–30% of the cases), other RAS/MAPK genes, such as *KRAS*, *SOS1*, *RAF1*, *KRAS*, *NRAS*, *BRAF*, and *MAP2K1*, are implicated, and during the last years, novel genes have been identified in association with NS, including *RIT1*, *RRAS*, *RASA2*, *A2ML1*, *SOS2*, and *LZTR1* (4, 5).

Characteristic dysmorphic facial appearance is evident in children and evolves with age, becoming quite undetectable in adulthood (6, 7), such that mildly affected cases may be misdiagnosed. More than 80% of affected individuals have congenital heart defects, particularly pulmonary valve stenosis, often of mild entity, followed by atrial septal defects and hypertrophic cardiomyopathy (8). Birth weight and length are normal, although more than half of individuals with NS experienced short stature during childhood because of apparent aberrations in growth hormone secretion/responsiveness (7). Puberty can be delayed with the mean age of pubertal onset of 14 years in boys and 13.5 years in girls, but it generally starts spontaneously (9–12). Despite the lack of evidence, female gonadal function seems not to be affected in NS. In male individuals, cryptorchidism is common, occurring in up to 80% of the cases (13), and male fertility has been reported to be decreased with oligospermia or non-obstructive azoospermia (14–16). Several previous studies recognized cryptorchidism as the main factor contributing to infertility (13, 15, 17, 18). However, recent evidence suggests that it could be the result of primary gonadal dysgenesis occurring during fetal development with intrinsic Sertoli cell defects with/without Leydig cell impairment (9, 11, 19). However, only a few studies have focused on gonadal function and fertility in NS patients.

In this article, we described a case of male infertility in a patient with NS due to a pathogenic variant of the *LZTR1* gene.

## Case description

A 35-year-old man was admitted to the Section of Andrology of Pisa University Hospital in September 2022 because of erectile dysfunction. He had a stable relationship with a female partner for 5 years. Sexual desire was present and spontaneous nocturnal/morning erections were reported. He declared that he has not used medications or smoked and did not engage in physical activity. The patient had no children.

He was born from a spontaneous pregnancy. His birth weight and length are normal, but he had a short stature in childhood. Thus, he underwent treatment with recombinant human growth hormone from 5 to 16 years old. The patient had a spontaneous onset of puberty at 14 years of age.

He suffered from congenital pulmonary valve stenosis, atrial septal defects, and mitral valve prolapse of mild entity, requiring periodic follow-up. Electrocardiography showed left-axis deviation and incomplete right bundle branch block.

Medical history also revealed incisor agenesis, chronic bilateral deep venous insufficiency, surgical correction of the right inguinal hernia at the age of 32 years, and removal of three nevi.

His elder sister was stillborn, and his parents died at the age of 52 and 64 years because of breast cancer and bowel infarction, respectively.

**Figure 1:**
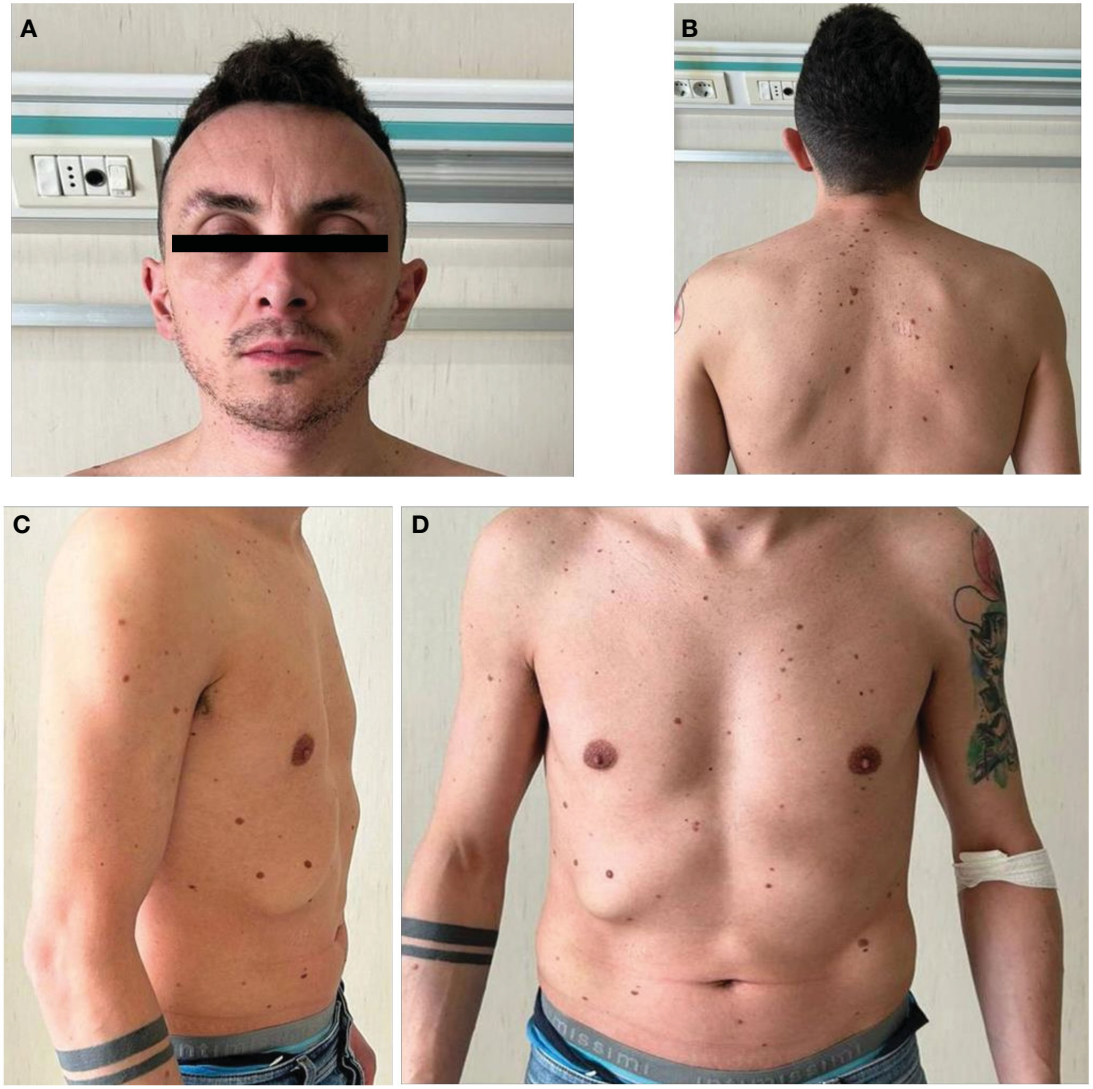
Facial and body appearance of the patient. Physical examination showed multiple nevi **(B–D)**, a triangle-shaped head **(A)** with a tall forehead and high anterior hairline, bilateral palpebral ptosis, low-set and posteriorly rotated ears, short neck with low posterior hairline **(B)**. The patient also showed chest deformity (superior pectus carinatum and inferior pectus excavatum) with widely spaced and low-set nipples **(C, D)**.

On clinical evaluation (**Figure 1**), the following data were recorded: height 1.73 m, weight 60 kg, and BMI 20 kg/m^2^. Multiple nevi were present. His facial features were characterized as follows: triangle-shaped head with a tall forehead and high anterior hairline, bilateral palpebral ptosis, low-set and posteriorly rotated ears, and short neck with low posterior hairline. He also had chest deformity (superior pectus carinatum and inferior pectus excavatum) with widely spaced and low-set nipples. Genital examinations showed reduced testis size with a volume of 10 ml (Prader orchidometer).

Recent hormonal evaluation showed a normal total testosterone level [22.2 nmol/L, normal range (n.r.) 8.7–29.1] with high serum FSH (19.3 IU/L, n.r. 1.5–12.4). Semen analysis (**Table 1**) documented severe oligospermia (sperm concentration 1.5 × 10^6^ per ml; total sperm number 6.2 × 10^6^ per ejaculate).

**Table 1:** Semen analysis (performed after 3 days of sexual abstinence).

Semen parameters	Patient data	Lower reference values^*^
Semen volume (ml)	4.2	1.4
Sperm concentration (10^6^ per ml)	1.5	16
Total sperm number (10^6^ per ejaculate)	6.2	39
Total motility (grade A + B + C, %)	38	42
Progressive motility (grade A + B, %) - Grade A motility (%) - Grade B motility (%) Non-progressive motility (grade C, %)	24 20 4 14	30 1
Immotile spermatozoa (grade D, %)	62	20
Normal forms (%) - Abnormalities of the head (%) - Abnormalities of the midpiece (%) - Abnormalities of the tail (%)	6 69 8 23	4

* Data refer to the 5th centile from Campbell et al. WHO (2021).

We repeated hormonal analysis that confirmed normal total testosterone level (19 nmol/L, n.r. 6.2–27), high FSH level (20.7 IU/L, n.r. 1.3–19.5), and normal LH level (4.2 IU/L, n.r. 1.4–12.7). AMH and inhibin B were low–normal (AMH 1.4 µg/L, n.r. 0.73–16; inhibin B 31 pg/ml, n.r. 25–325). Thyroid function and prolactin were normal. Insulin-like growth factor 1 was in the normal range (138.5 µg/L, n.r. 85–256). Laboratory results are reported in **Table 2**. Baseline coagulation screening was normal.

**Table 2:** Laboratory results.

Parameters	Patient data	Reference values
LH (IU/L)	4.2	1.4–12.7
FSH (IU/L)	20.7	1.3–19.5
Total testosterone (nmol/L)	19	6.2–27
AMH (μg/L)	1.4	0.7–16
Inhibin B (pg/ml)	31	25–325
PRL (μg/L)	13.9	2–13
TSH (mIU/L)	1.32	0.4–4
IGF-1 (μg/L)	138.5	85–256

Testicular ultrasound confirmed reduced testes volume (right testis volume 9 cc and left testis volume 6 cc) without focal lesions. Abdomen ultrasonography excluded hepatosplenomegaly or renal anomalies.

**Figure 2:**
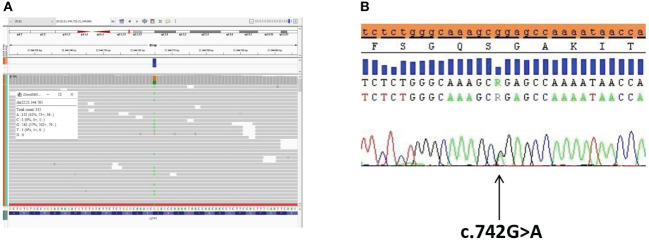
The *LZTR1* variant identified by exome sequencing analysis and confirmation via Sanger sequencing. **(A)** The *LZTR1* variant visualized by the Integrative Genomics Viewer (IGV) (reference base G in orange, variant base A in green) in the proband. **(B)** Sanger sequencing analysis of the *LZTR1* variant visualized by SeqScape in the proband.

We performed next-generation sequencing (NGS) of the genes involved in the RAS/MAPK pathway (the custom panel having 19 genes including the coding sequence and the donor and acceptor splice sites: *PTPN11*, *SOS1*, *SOS2*, *RAF1*, *BRAF*, *CBL*, *KRAS*, *NRAS*, *HRAS*, *RIT1*, *LZTR1*, *SPRED1*, *SHOC2*, *MAP2K1*, *MAP2K2*, *MRAS*, *PPP1CB*, *RASA2*, *RRAS2*). NGS was performed through Nextera Flex IDT-custom panel (Illumina-IDT) in paired-end mode 2 × 150 using the MiSeq (Illumina) platform. Sequencing analysis demonstrated the presence of the pathogenic heterozygous variant c.742G>A, p.(Gly248Arg) of the *LZTR1* gene (NM_006767.3). The variant was validated by Sanger sequencing (**Figure 2**). The karyotype was normal (46,XY), and no Y chromosome microdeletions in azoospermia factor (AZF) regions were found.

### Patient consent

Written informed consent for the publication of the article and accompanying clinical images was obtained from the patient.

## Discussion

NS is a complex and relatively common disorder that affects multiple organ systems with variable severity, caused by variants in the RAS/MAPK pathway. The phenotype is well characterized in childhood, with distinctive facial features and organ abnormalities; however, it is more subtle in adulthood which is often misdiagnosed or not detected by clinicians.

In this article, we reported the case of a 35-year-old man with no previous diagnosis of NS, who was referred to us because of andrological concern (erectile dysfunction and severe oligospermia). The other typical abnormalities associated (mainly congenital pulmonary valve stenosis and other cardiac anomalies and a history of short stature) allowed us to suppose that it was an NS, which was subsequently confirmed by genetic analysis. Clinical features were very mild but still visible. Typical ECG pattern (20), dental anomalies (incisor agenesis), multiple lentigines and nevi, and chronic bilateral deep venous insufficiency supported the diagnosis. Andrological history/examination revealed delayed puberty from 14 years with low testes volume and previous surgical correction of inguinal hernia. The patient’s biochemical profile showed high serum FSH levels associated with low–normal AMH and inhibin B values, compatible with primary Sertoli cell defects. No cryptorchidism was reported at birth, and the levels of testosterone were normal, reflecting normal Leydig cell function.

Gonadal dysfunction and subfertility are rarely investigated in these subjects, and the underlying mechanisms are still incompletely understood. Several articles report that gonadal dysfunction in NS is secondary to cryptorchidism (which is present at birth in the majority of cases) and consequent testes damage (13, 15, 17, 18, 21). However, recent evidence suggests that gonadal dysfunction is a feature of the syndrome and is rather related to abnormal testes development (9, 11, 19, 22). Gonadal impairment can also affect NS individuals with normal testicular descent (19, 22, 23). Moreover, genital tract defects other than cryptorchidism, such as hypoplastic external genitalia, penile hypospadias, and inguinal hernia, have been reported in NS individuals (24–26). Primary gonadal dysfunction is supported by the demonstration that the *PTPN11* gene (which variant is the most implicated in the syndrome) is expressed in germ cells and Sertoli cells and has a pivotal role in the regulation of spermatogenesis (27–29). As for other clinical manifestations of NS, as well as for Sertoli cell dysfunction, a genotype/phenotype correlation has been observed (9). Aside from patients with the *PTPN11* variant, significantly low levels of Sertoli cell markers (AMH and inhibin B) were found by Moniez et al. in two patients with *RAF1* variants (23), while Marcus et al. showed Sertoli cell dysfunction (raised FSH level in combination with low inhibin B concentration) in one *BRAF* patient with normal testicular descent (22). *SHOC2* and *KRAS* variants were associated with delayed puberty and/or low testicular volume in the study of Patti et al., but no information on fertility was available in these patients (11). In the other cases of male gonadal dysfunction, genetic characterization was lacking or only the *PTPN11* gene was tested.

In our case report, we identified the pathogenetic heterozygous variant c.742G>A, p.(Gly248Arg) of the leucine-zipper-like transcriptional regulator 1 (*LZTR1*) gene [a rare variant that accounts for approximately 2%–3% of NS cases (30)].

The *LZTR1* gene is a tumor suppressor that encodes a protein member of the BTB-kelch superfamily, located within the 3-Mb-long region. Its function is lacking, although Nacak et al. (31) characterized the LZTR1 protein on the cellular level as a BTB-kelch protein that exclusively localizes to the Golgi complex (maybe to stabilize it). The LZTR1 protein has been only recently associated with the RAS–MAPK signaling pathway; Motta et al. (32) proposed a model in which LZTR1 negatively modulates this pathway; consequently, pathogenetic variants that inactivate LZTR1 result in a RAS–MAPK signaling upregulation. *LZTR1* variants can be associated with either the dominant or recessive form of NS; the variants associated with dominant NS are located in the highly conserved kelch domain, while the variants associated with recessive forms have been found throughout the protein (2, 3, 33).

The detected variant c.742G>A, p.(Gly248Arg) of the *LZTR1* gene results in a non-conservative amino acid change located in the Kelch repeat type 1 of the encoded protein sequence and has been reported in the literature in individuals affected with NS (including familial and *de-novo* cases). Yamamoto et al. (34) identified the variant in a cohort of NS probands with typical facial features (downslanting palpebral fissures, hypertelorism, ptosis, short/webbed neck) and cardiac abnormalities (pulmonary stenosis), but not short stature and cognitive disabilities; furthermore, one individual of the cohort developed multiple schwannomas. Chinton et al. (33) detected the same variant in five NS patients with heart defects, neurodevelopmental delay, and short stature and described acute lymphoblastic leukemia in one subject. We had no notice about gonadal function and fertility status in these cohorts of NS patients. Umeki et al. (25) reported the variant in a child with NS who showed pulmonary stenosis, anomalous origin of the coronary artery, and a concealed penis but no cryptorchidism. Finally, Güemes (35) identified the variant, which appeared *de novo*, in a boy with typical dysmorphic features, cardiac abnormalities, and bilateral cryptorchidism. **Table 3** summarizes the clinical manifestations of the reported cases of NS from the heterozygous *LZTR1* variant c.742G>A, p.(Gly248Arg).

**Table 3:** List of case reports of male individuals with the heterozygous *LZTR1* variant c.742G>A, p.(Gly248Arg): clinical findings.

Authors	Dysmorphic features	Short stature	Congenital heart defects	Neurodevelopmental delay	Genital defects	Tumors
Yamamoto et al., 2015 (30)	Yes	No	Yes (PS, ASD)	No	N/A	Multiple schwannomas
Chinton et al., 2019 (33)	Yes	Yes	Yes (ASD)	Yes	N/A	Acute lymphoblastic leukemia
Umeki et al., 2019 (25)	Mild	No	Yes (PS, anomalous origin of the coronary artery)	No	Concealed penis	No
Güemes et al., 2019 (35)	Yes	No	Yes (PS)	No	Bilateral cryptorchidism	No
Our case report	Mild	Yes	Yes (PS, ASD, MVP)	No	Hypoplastic testes, oligospermia, ED, IH	No

PS, pulmonary stenosis; ASD, atrial septal defect; MVP, mitral valve prolapse; ED, erectile dysfunction; IH, inguinal hernia; N/A, not available.

Hence, although gonadal function was not available in most of the cases, we could suppose that, similar to *PTPN11*, also the *LZTR1* gene has a role in testicular development and spermatogenesis. More studies are necessary to define the exact role of the *LZTR1* gene in germ cell development. However, fertility status assessment should be performed in NS individuals with *LZTR1* variants, and andrological evaluation should be offered to the affected individuals. Furthermore, acting like a tumor-suppressor gene, NS individuals with *LZTR1* variants need a more careful follow-up because of the potential higher risk of malignant tumors.

In conclusion, NS is a relatively common disorder, and the characteristic features, although subtle in adulthood, should induce clinicians to suspect the syndrome and to propose a genetic test. Variants in the *LZTR1* gene are rare, and their manifestation is still not clearly delineated because of the few reported cases. This case report enriches the clinical spectrum of *LZTR1* variants that seem to be associated with the complete phenotype of the syndrome (facial dysmorphology, cardiac defects, short stature), including male semen abnormalities. Finally, it provides increased knowledge on reproductive disorders affecting male NS individuals, focusing on primary gonadal dysfunction and subfertility as part of the syndrome.

## Ethics statement

Written informed consent was obtained from the individual(s) for the publication of any identifiable images or data included in this article.

## Author contributions

FO: Writing – original draft, Methodology, Investigation, Data curation, Conceptualization. LP: Writing – original draft. FB: Writing – review & editing, Visualization, Data curation, Conceptualization. SR: Writing – review & editing, Visualization. MT: Writing – review & editing, Visualization, Supervision. DC: Writing – review & editing, Visualization, Validation, Supervision.
